# Current efforts towards safe and effective live attenuated vaccines against African swine fever: challenges and prospects

**DOI:** 10.1186/s40249-021-00920-6

**Published:** 2021-12-24

**Authors:** Tao Wang, Rui Luo, Yuan Sun, Hua-Ji Qiu

**Affiliations:** 1 State Key Laboratory of Veterinary Biotechnology, Harbin Veterinary Research Institute, Chinese Academy of Agricultural Sciences, Harbin, 150069 China; 2 School of Life Science Engineering, Foshan University, Foshan, 528231 China

**Keywords:** African swine fever, Live attenuated vaccine, Efficacy, Safety, Differentiation of infected from vaccinated animals

## Abstract

**Background:**

African swine fever (ASF) is a fatal hemorrhagic disease in domestic pigs and wild boar caused by African swine fever virus (ASFV). Since ASF has been introduced into Europe and Asia, the major pig-raising areas, posing a huge threat to the pork industry worldwide. Currently, prevention and control of ASF are basically dependent on strict biosecurity measures and stamping-out policy once ASF occurs.

**Main text:**

The major risks of ASF spread are insufficient biosecurity measures and human behaviors. Therefore, a safe and effective vaccine seems to be a reasonable demand for the prevention and control of ASF. Due to the efficacy advantage over other types of vaccines, live attenuated vaccines (LAVs), especially virulence-associated genes deleted vaccines, are likely to be put into emergency and conditional use in restricted areas if ASF is out of control in a country with a huge pig population and pork consumption, like China. However, the safety, efficacy, and genetic stability of current candidate ASF LAVs require comprehensive clinical evaluations prior to country-wide field application. Several critical issues need to be addressed to commercialize an ideal ASF LAV, including a stable cell line for manufacturing vaccines, differentiation of infected from vaccinated animals (DIVA), and cross-protection from different genotypes.

**Conclusion:**

A safe and effective DIVA vaccine and an accompanying diagnostic assay will facilitate the prevention, control, and eradication of ASF, which is quite challenging in the near future.

**Graphical Abstract:**

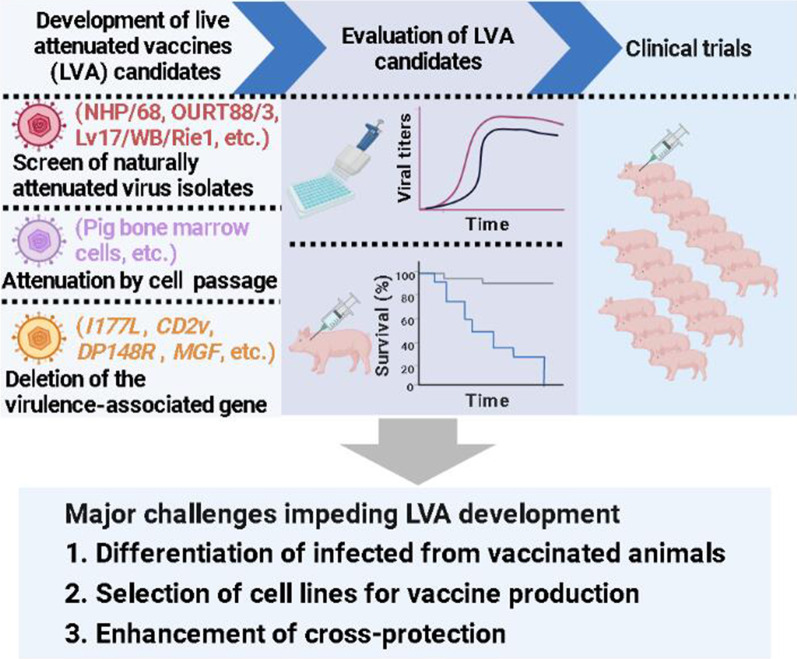

## Background

African swine fever (ASF) is a widespread hemorrhagic and fatal disease caused by ASF virus (ASFV) that occurs in domestic pigs and wild boar [1]. ASF was first discovered in 1921 in Kenya, and it has been endemic in many countries in Africa [2]. In 1957, ASF was introduced for the first time to the Iberian Peninsula and had circulated for more than 30 years. The disease severely struck the local pig industry and was not eradicated until 1996 (except for Sardinia) through biosecurity measures and eradication programs. In 2007, ASF was reintroduced from Africa to Europe (the Caucasus), spreading quickly to most Eastern European countries. The situation was worsened considerably as ASF spread to China in 2018 and quickly spread to 11 Asian countries [3]. Moreover, ASF spread to some European Union member states, including Belgium, the Czech Republic, Romania, Bulgaria, and Germany. Since no commercial vaccine is currently available, the prevention and control of ASF are essentially based on biosecurity and rapid test-removal policy. The pandemic of ASF has caused significant economic losses and threatened global pork production.

ASFV is a member of the family Asfarviridae (African swine fever and related viruses) and is the only known DNA arbovirus [1, 5]. The ASFV genome is a double-stranded DNA molecule of 170–194 kb that contains 150–167 open reading frames (ORF), depending on the virus strains [1, 4]. Based on the nucleotide differences at the end of the *B646L* (p72) gene, 24 ASFV genotypes have been identified [5]. The lack of safe and effective ASF vaccines is a big gap in the prevention and control of ASF [5]. Compared with other types of ASF vaccines, live attenuated vaccines (LAVs) can provide complete homologous and partial heterologous protection, representing a promising and feasible vaccine strategy in the foreseeable future [6]. With further safety improvement, LAVs are likely to be put into emergency and conditional use in restricted areas if ASF emerges or reemerges or is out of control in a country with a huge pig population and pork consumption, like China and the USA.

## Strategies for generating ASF LAV candidates

### Screen of naturally attenuated virus isolates

Naturally attenuated (NA) strains with reduced virulence and/or non-hemadsorbing (non-HAD) will occur during the natural epidemic of ASFV [7–11]. Notably, the non-HAD genotype I ASFV strains NH/P68 and OUR T88/3 were isolated from chronically infected pigs and soft ticks, respectively, when ASFV was circulating in the Iberian Peninsula [7, 8]. Pigs that survived infection with the NH/P68, or OUR T88/3, can protect against the virulent ASFV strain infection, indicating that the NA ASFV strain has the potential to be developed as a LAV. However, some side effects were also reported, including necrotic skin lesions and the swelling of joints, emphasizing that NA ASFV strains have residual virulence. Gallardo et al. isolated a non-HAD genotype II NA ASFV strain, Lv17/WB/Rie1, from wild boar in 2017 [10]. It can protect wild boar from challenge with the virulent ASFV Arm07 strain [10, 11]. Nevertheless, the safety, virus shedding, and genetic stability of Lv17/WB/Rie1 need to be further evaluated. Two years after the ASF outbreak in China, low virulent ASFV strains were isolated in 2020 [12]. Due to residual virulence, the NA strains are unlikely to be developed as LAVs. Further deletion of virulence-associated genes (VAG) based on NA candidates may improve the safety of LAVs.

### Attenuation by cell passage

In addition to NA ASFV isolates, the continuous passage of a virulent ASFV strain in heterologous cells or hosts is a feasible strategy to develop LAVs. When ASF was first introduced into Europe, the researchers found that the virulent ASFV strains were attenuated after the continuous passage in porcine bone marrow or kidney cells [13, 14]. Subsequent challenge experiments showed that the pigs immunized with the attenuated strain were protected against virulent strain infection. Although the attenuated strains showed a great promise in laboratory evaluation, the results of the field trials were disappointing [13]. Using a similar strategy, the virulent ASFV strain Stavropol 01/08 was attenuated by passages in a hybrid cell line A_4_C_2_/9k. The ASFV of passages 24 and 33 in A_4_C_2_/9k cells and passage 20 in CV-1 cells lost pathogenicity to pigs but did not protect pigs against virulent strain infection [15]. The virulent genotype II ASFV Georgia strain (ASFV-G) lost pathogenicity and immunogenicity after 110 passages in Vero cells [16]. As mentioned above, the immunogenicity and efficacy of the cell-adapted LAVs differ significantly and need to be evaluated in pigs.

A recent study reported that the ASF vaccine strain ASFV-G-ΔI177L/ΔLVR, which was generated by adaptation of the ASFV-G-ΔI177L strain to the porcine fetal kidney cell line (PIPEC), displayed attenuation and protective efficacy similar to ASFV-G-ΔI177L [17]. Although this discovery brings hope to the large-scale production of the ASF vaccine in cell lines, the safety and genetic stability of the ASFV-G-ΔI177L/ΔLVR strain still needs further evaluation. Furthermore, we recently demonstrated that ASFV adapted to HEK293T cells with deletion of the left-end variable MGF genes [18]. The deletion of *MGF300* (*1L*, *2R*, and *4L*) and *MGF360* (*8L*, *9L*, *10L*, and *11L*) genes might be important for the adaptation of ASFV to HEK293T cells at the early stage [18]. Moreover, the pathogenicity and immunogenicity of the HEK293T cell-adapted ASFV will be evaluated in pigs. In the future, the NA ASFV strains or VAG deleted attenuated vaccine candidate strains can be adapted to cell lines, which are expected to improve the safety profile and facilitate the large-scale production of LAVs.

### Deletion of the virulence-associated gene(s)

The precise deletion VAG of virulent strain to construct LAV candidates has become a critical approach to current ASF LAVs research. Compared with isolation of NA strains and attenuation of virulent strain by cell passage, individual or combined deletion of VAG can attenuate virulent ASFV isolate and induce protective immune responses against virulent parental virus challenge in pigs (Table 1). A recombinant LAV strain BA71ΔCD2v, lacking the *CD2v* gene, can confer protection against homologous or heterologous ASFV infection [19]. A further study showed that 83.3% and 33.3% of the pigs immunized with 10^6.0^ PFU BA71∆CD2 survived when challenged with the genotypes XIX and IX ASFV, respectively [20]. These results confirmed that cross-protection is a multifactorial phenomenon that not only depends on sequence similarity [20]. The genotype II ASFV LAV strain ASFV-G-ΔI177L, constructed by deleting the *I177L* gene from the virulent strain ASFV-G, has been proven safe and highly efficacious in challenge studies [21]. Further research has shown that the ASFV-G-ΔI177L vaccine candidate can be administered by oronasal (ON) route, which can achieve similar efficacy to intramuscular (IM) administration [22].

**Table 1:** Promising progress towards the development of live attenuated vaccines against African swine fever

Parental strains	Genotypes	Attenuated strategies	LAV candidates	Protection	Production cells	References
NH/P68	I	Naturally attenuated	NH/P68	Homologous strain (L60, Arm07)	PBMs	[7]
OURT88/3	I	Naturally attenuated	OURT88/3	Homologous strain (OURT88/1, Ug65)	BMs	[8]
Lv17/WB/Rie1	II	Naturally attenuated	Lv17/WB/Rie1	Homologous strain (Armo7)	PBMs	[10, 11]
BA71	I	DVAG (CD2v)	BA71ΔCD2v	Homologous and heterologous strain (E75, Georgia 2007)	COS-1	[19]
HLJ/18	II	DVAG (MGF505-1R, MGF360-12L, MGF360-13L, MGF360-14L, MGF505-2R, MGF505-3R, and CD2v)	HLJ/18-7GD	Homologous strain (ASFV HLJ/18)	PAMs	[23]
Georgia 2007	II	DVAG (I177L)	ASFV-G-ΔI177L	Homologous strain (Georgia 2007)	PAMs	[21]
ASFV-G-ΔI177L	II	DVAG (I177L) and cell passage	ASFV-G-ΔI177L/ΔLVR	Homologous strain (Georgia 2007)	PIPEC	[17]
ASFV-SY18	II	DVAG (CD2v and UK)	ASFV-SY18-ΔCD2v/UK	Homologous strain (ASFV-SY18)	PAMs	[24]
ASFV-SY18	II	DVAG (I226R)	SY18ΔI226R	Homologous strain (ASFV-SY18)	PAMs	[25]
Georgia 2010	II	DVAG (A137R)	ASFV-G-ΔA137R	Homologous strain (Georgia 2010)	PAMs	[26]

*DVAG* deletion of virulence-associated genes; *PBMs* porcine blood monocyte/macrophages; *BMs* pig bone marrow cells; *COS-1* monkey kidney tissue-derived cells; *PAMs* primary porcine alveolar macrophages; *PIPEC* Plum Island porcine epithelial cells (PIPEC), a porcine fetal kidney cell line engineered to express the bovine αVβ6 integrin; *LAV* Live attenuated vaccine

A Chinese research group generated a series of VAGs-deleted viruses based on the genotype II virulent strain ASFV HLJ/18, among which the mutant HLJ/18-7GD (lacking the *MGF505-1R*, *MGF360-12L*, *MGF360-13L*, *MGF360-14L*, *MGF505-2R*, *MGF505-3R,* and *CD2v* genes) was evaluated to be safe and protective in specific-pathogen-free (SPF) pigs [23]. Subsequent field trials in fattening pigs, sows, and wild boar showed that HLJ/18-7GD was genetically stable, unlikely to cause complications, and converted to virulence following vaccination. Our group showed that a genotype II ASFV mutant lacking the *CD2v* and *UK* genes was attenuated and elicited effective immune responses in pigs, providing complete protection against the challenge with the parental ASFV strain [24]. Although some new VAGs (e.g. *I177L*, *I226R*, and *A137R*) have been identified, the underlying mechanisms by which these genes affect virulence remain unclear [21, 25, 26]. The combined deletion of VAGs may over-attenuate ASFV and lead to a decrease or loss of protection [27, 28]. In the next 3 to 5 years, strengthening the functional research of VAGs and identifying new VAGs will accelerate the rational engineering of ASF LAVs with improved safety profiles. However, the delicate balance between the attenuation and immunogenicity of ASFV is a major challenge for the development of ASF LAVs.

### Evaluation of LAV candidates

**Fig. 1:**
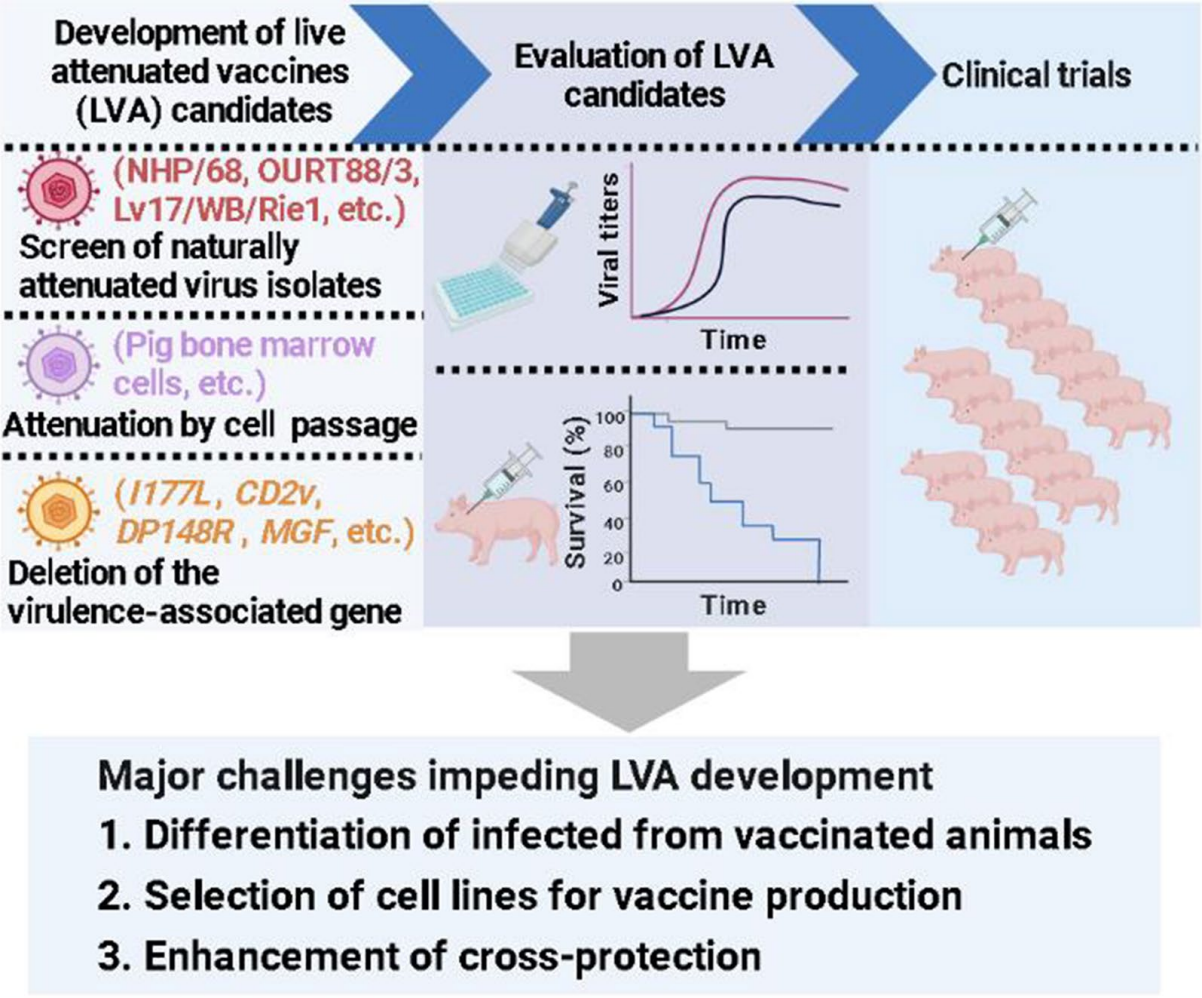
Development of live attenuated vaccines against African swine fever (created with BioRender.com)

After generating ASF LAV candidates through the above three strategies, it is necessary to conduct in vivo evaluation in biosafety level 3 (BSL-3) facilities to identify a safe and effective LAV candidate and then undergo a large-scale clinical evaluation to determine whether the vaccine candidate can be commercialized (Fig. 1). The in vivo evaluation procedure for the virulence and safety of LAV candidates is to use different doses (e.g. 10^2.0^ − 10^7.0^ HAD_50_) to inoculate pigs and observe the clinical signs including fever parameters, anorexia, recumbence, skin hemorrhage, or cyanosis, joint swelling, respiratory distress, and ocular discharge for 14–28 days [19–23]. Oropharyngeal, rectal excretion, and blood samples were collected at different days post-infection (dpi) to detect virus shedding, viremia, antibodies, and cellular immune response. Most of the general procedures for immunogenicity and protective efficacy evaluation via IM or ON route with 10^2.0^–10^7.0^ HAD_50_ LAV candidate and challenge with a lethal dose of parental strain at 14–28 dpi, or after boost immunization with the same route on 14–28 dpi [29, 30]. According to current research, viremia after vaccination is a feature of most ASF LAVs, effectively protecting pigs against virulent ASFV challenges [17]. Few studies have used heterologous ASFV strain to evaluate cross-protection efficacy. Due to the multiple factors and the genetic diversity of ASFV that affect the evaluation results of LAV, it is necessary for global ASFV research experts to jointly draft and formulate guidelines for the trials of ASF LAVs, which will help to improve the reliability of the evaluation results.

### Major challenges impeding LAV development

Currently, the commercialization prospect of ASF LAVs is not clear. In addition to the positive results, also have many safety issues, virus shedding, development of post-vaccination complications, and insufficient protection of immunocompromised pigs. As discussed above, most ASF LAVs are evaluated through short immunization protocols, which is far from enough to evaluate the safety of LAVs. This is due to the insufficient market demand for ASF vaccines before 2018, and the evaluation tests of LAVs need to be carried out in BSL-3 facilities. Presently, the global epidemic of ASF has increased the market demand for the vaccines. The governments and enterprises have expanded funding to support ASF vaccine development. Therefore, it is necessary to evaluate the duration of protection of current LAV candidates [31]. This evaluation can provide reliable data support for clinical trials and help explore the molecular mechanism of vaccine-induced protective immunity and design a safe and effective vaccine against ASF.

The continuous passage of LAVs in pigs to assess the risks of conversion to virulence is very important for ensuring the safety of ASF LAVs. Infection with attenuated strains of different genetic backgrounds to assess the genetic recombination is also essential for evaluating the safety of ASF LAVs. These evaluations can determine whether ASF LAVs are safe enough to ensure that it can be used in the field. In addition, there are no adequate evaluations for the horizontal transmission of ASF LAVs and the risks of vertical transmission of sows after immunization. Therefore, it is still controversial whether the safety of candidate strains of ASF vaccines meets the requirements for application in the field.

When the safety and efficacy requirements are met, the first-generation ASF LAVs may be used under certain conditions, such as all-in and all-out fattening pig farms, pig farms in areas threatened by ASF outbreaks [32]. However, several problems still need to be solved for next-generation LAVs. Most LAVs are grown on PAMs, and no stable cell lines are available for manufacturing vaccines [29–32]. It has been reported that BA71ΔCD2v can replicate in COS-1 cells, ASFV-G-ΔI177L can replicate in PIPEC cells after adaptation. However, the genome stability and immunogenicity maintenance of ASF LAVs after continuous passage in the abovementioned cells still need many assessments. ASF LAVs have limited cross-protective efficacy. It is still a considerable challenge to develop an LAV with cross-protective efficacy against genotypes I and II virulent ASFV strains circulating in Eurasia.

### Detection technologies accompanying the gene-deleted ASF vaccines

Developing a detection technology to differentiate infected from vaccinated-animal (DIVA) is essential for prevention, controlling, and eradicating ASF. There are two DIVA strategies for the ASF LAVs. One is to establish a multiplex real-time PCR targeting the *p72* gene of the wild-type ASFV strain and the deleted gene(s) of the ASF LAVs, respectively. The other is to detect antibodies induced by the deleted gene-encoded protein by enzyme-linked immunosorbent assay (ELISA). At present, based on CRISPR/Cas12a-based nucleic acid detection technology, recombinase polymerase amplification and gold nanoparticles technology have been reported for the rapid detection of ASFV. However, its commercial use still needs a particular time [33–36]. Currently widely used detection technology is the real-time PCR targeting the *p72* gene, which shows high sensitivity and specificity. NA ASFV strains could emerge after several years in the field, which induce inapparent or mild clinical signs with longer incubation time, thus the detection of anti-ASFV antibodies will be required for surveillance. In general, the development and commercialization of more sensitive, convenient, high-throughput, and easily accessible etiological and serological technologies are essential for monitoring and eradicating ASF.

## Conclusions

A safe and effective vaccine and sensitive, specific, high-throughput, and readily available detection technologies are the basis for preventing, controlling, and eradicating ASF. They are also issues that the scientific community urgently needs to solve. In the near future, regardless of the success or failure of the scientific community to develop a safe and effective ASF vaccine, the control of ASF always requires prevention and control based on the principles of biosafety and rapid diagnosis.

## Data Availability

Not applicable.

## Abbreviations

ASF: African swine fever
ASFV: African swine fever virus
ORFs: Open reading frames
LAV: Live attenuated vaccine
NA: Naturally attenuated
non-HAD: Non-hemadsorbing
VAGs: Virulence-associated genes
ON: Oronasal
IM: Intramuscular
BLS-3: Biosafety level 3
DIVA: Differentiation of infected from vaccinated animals
DPI: Days post-infection
PAMs: Primary porcine alveolar macrophages
ELISA: Enzyme-linked immunosorbent assay
